# Subfascial Breast Augmentation: A Systematic Review and Meta-Analysis of Capsular Contracture

**DOI:** 10.1093/asjof/ojaa006

**Published:** 2020-02-05

**Authors:** Daniel J Gould, Orr Shauly, Levonti Ohanissian, W Grant Stevens

**Affiliations:** 1 Department of Plastic and Reconstructive Surgery, Keck Hospital, University of Southern California, Los Angeles, CA; 2 Keck School of Medicine, University of Southern California, Los Angeles, CA; 3 Miller School of Medicine, Miami, FL; 4 Clinical Professor of Surgery and Director of the Aesthetic Surgery Fellowship, Division of Plastic and Reconstructive Surgery, School of Medicine, University of Southern California, Los Angeles, CA

## Abstract

**Background:**

Subfascial breast augmentation is a technique originally developed to reduce the risks of capsular contracture while decreasing the postoperative pain associated with subpectoral augmentation. It was pioneered in Brazil by Dr. Graf and others, and recently this technique has gained interest in the aesthetic world.

**Objectives:**

The goal of this study was to provide a systematic analysis of subfascial breast augmentation to assess the combined reported rates of capsular contracture, animation deformity and complications.

**Methods:**

The PubMed, Embase, and Web of Science databases were searched for the use of the subfascial plane for breast augmentation. We included studies that reported on capsular contracture and other outcomes following subfascial breast augmentation.

**Results:**

Through the initial search, 26 articles were identified. Of which, 22 were included in the final study. A total of 3743 patients were identified across these studies with a total number of 38 cases of capsular contracture representing a rate of 1.01% of capsular contracture. Several articles reported on demographics, perioperative and long-term complications, and outcomes with regards to the aesthetic outcome from the surgeon’s perspective. Several infections were reported representing a rate of 0.1%. Animation deformity was not reported, although rippling was occasionally reported as was malrotation, axillary banding, sensory deficit, and asymmetry. Subfascial breast augmentation appears to have a low complication rate and an extremely low rate of capsular contracture at approximately 1%.

**Conclusions:**

Subfascial breast augmentation may provide the benefits of low rates of capsular contracture while avoiding the discomfort and future animation deformity of subpectoral augmentation.

**Level of Evidence: 4:**

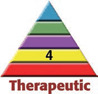

Subfascial breast augmentation utilized placement of the breast implant below the prepectoral fascia and above the pectoral muscle through either an inframammary, transaxillary, or periareolar incision.^1-4^ The technique was developed to combine the benefits of the subfascial plane including decreased rates of capsular contracture and blunting of the implant edges and the creation of an anatomic pocket for precise implant placement while reducing the pain from submuscular pocket dissection as well as animation deformity.^5-7^ Subfascial augmentation has been previously recommended for thin or athletic patients, as the intact fascia can blunt the edges and reduce the visibility of the implant and it does not disrupt the pectoral muscle.^8-10^ It has been described as a composite breast augmentation in conjunction with fat grafting as well as a technique for thin patients who wish to camouflage the implant edge.^11^

Classical efforts have been focused at blunting the step off in the superior pole through the use of the subpectoral pocket or with a dual plane approach.^12^ Patients may have excellent outcomes with this type of reconstruction, but there may be less pain and spasm with subfascial implant placement. Several studies have sought to elucidate advantages and have suggested subfascial implantation may have some benefits compared to submuscular.^6,8,13-15^ Furthermore, several large case series have reported long-term outcomes and very low rates of capsular contracture.^11,16-19^ There have been reported cases of capsular contracture and methods to correct capsular contracture in patients with subfascial augmentation.^20^

Given these reports, the goal of this study was to examine the literature on subfascial augmentation to determine outcomes with regards to capsular contracture, animation deformity, and complications. Study participants included in the literature are largely undefined; however, a comparison of outcomes is still warranted. A systematic review of the literature has not yet been performed on this subject. As such, rates of capsular contraction among those patients that have received subfascial breast augmentation will be investigated in relation to subglandular and submuscular augmentation.

## METHODS

The primary endpoint of this review was to study the effect of subfascial breast augmentation on capsular contracture and other complications, including revisions, hematoma, seroma, infection, animation, ripples, malrotation, and asymmetry. Secondary analysis was conducted to determine the effect of subfascial breast augmentation on aesthetic outcome. The patient follow-up period and demographics were also evaluated.

### Study Selection

**Figure 1.**
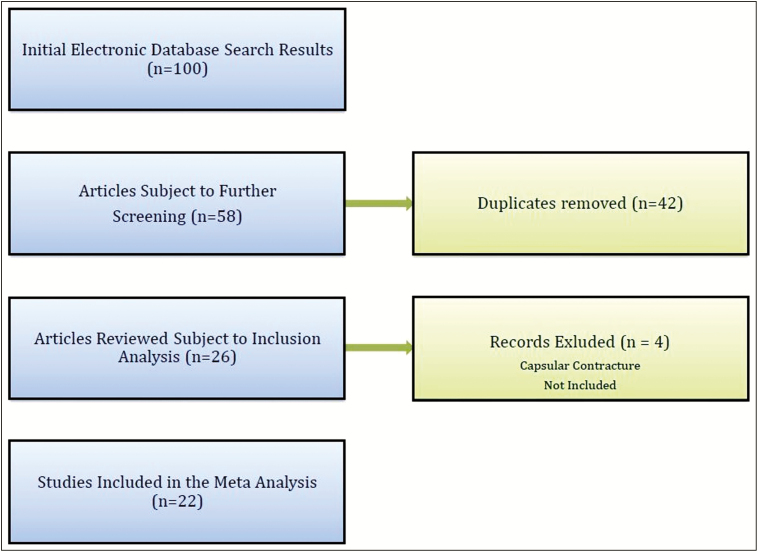
Flowchart depicting the stepwise approach for the identification of studies of capsular contraction in subfascial primary augmentation to include in the meta-analysis.

On April 9, 2017, we conducted a search of published articles in PubMed, Embase, and Web of Science databases. The search was done with no restrictions and for specific search terms: “subfascial, retrofascial, breast augmentation, mammaplasty.” After excluding duplicates, the authors (L.O. and D.G.) screened the titles and abstracts of all the articles. Studies evaluating subfascial breast augmentation and capsular contracture were considered candidate studies and the full text of each of these articles was assessed for further evaluation based on the inclusion and exclusion criteria established prior to the literature search. In cases of disagreement between the two screeners, another author (O.S.) assessed the full text and resolved the dispute. If there was further disagreement, the senior author was consulted. The reference list of each study was manually screened for additional pertinent articles (Figure 1).

To be included in the meta-analysis, a published study had to measure capsular contracture in patients who have undergone primary breast augmentation in the subfascial plane only. Studies between the years 2000 and 2017 were included given that modern developments of the technique have largely occurred in the 21st century.

Excluded from the meta-analysis were studies that included patients who underwent breast augmentation only partially in the subfascial plane, studies that focused on most appropriate implant type and not complications of subfascial breast augmentation, and studies that only included reoperations albeit in the subfascial plane.

### Data Collection

Data from each study were extracted into a form with the following parameters: publication citation, number of patients, number of capsular contracture, baker class, number of hematomas, infections, revisions, seromas, ripples, scarring, sensory deficits, malrotations, asymmetries demographic information, and patient satisfaction criteria.

### Meta-Analysis

After collecting 22 studies with reported capsular contracture rates, the studies were subjected to a meta-analysis to determine the overall rates of capsular contracture and the weighted effects of each study. Several methods were utilized toward this end^21-33^ and Microsoft Excel was used for calculation of the effects, statistical analysis of the studies as well as the visual display of the data in the form of forest plots with the reported outcomes. The overall heterogeneity was reported and a Q-test was applied as well as a calculation of *I*^2^ which is an indicator of heterogeneity previously reported in the literature.^27^ Models under the assumptions of either fixed or random effects were also studied. The principle summary measure is a difference in pooled means. Risk of bias was not assessed for the observational studies included, since a majority of the STROBE checklist could not be evaluated from the publicly available text. The quality of this systematic review and meta-analysis was assessed using the PRISMA checklist (Supplementary Appendix A).

## RESULTS

Initial electronic database search resulted in 100 manuscripts. After further screening of title and abstract, 58 articles were subject to further screening. Following inclusion and exclusion analysis, a total of 22 articles representing data on 3743 patients was included in this review and meta-analysis.

### Demographics

Unfortunately, very little was reported in the qualifying studies on the actual demographics of the patients receiving implants. Though many studies stated that these patients were similar to other patients seeking primary augmentation, there were few studies with reported age range, BMI, or other medical information about the augmented patients (Table 1). However, there were large numbers of patients in each of the study groups, with a total of 3743 patients reported across all included studies.

**Table 1.** Patient Demographics of Included Studies

Study	Demographics	No. of patients who underwent subfascial BA
Araco, 2007	NR	511
Aygit, 2013	Ages ranged from 19 to 32 years, with a mean of 27.3 years	27
Barbato, 2004	Ages ranged from 15 to 55 years, and the most frequent age period was 15 to 24 years (33.5% of the study population)	110
Benito-Ruiz, 2003	NR	16
Brown, 2012	NR	200
Goes, 2003	NR	241
Graf, 2000	Ages 15 to 48 years (for all subjects including those who did not undergo subfascial augmentation)	8
Graf, 2003	NR	263
Graf, 2005	NR	415
Hunstad, 2010	NR	61
Keramidas, 2006	NR	350
Kerfant, 2017	Ages ranged from 19 to 51 years, with a mean of 31.7 years. Average body mass index was 18.85 kg/m^2^	156
Lin Jinde, 2010	Patients with small or moderate breasts ages from 23 to 43 years	10
Munhoz, 2006	Ages from 18 to 37 years (mean, 26 years). All patients underwent primary breast augmentation under general anesthesia as outpatients. The types and sizes of the implants (textured silicone gel implants) varied from 210 to 305 ml in size.	42
Pereira, 2009	Ages ranged from 18 to 29 years, with a mean of 25.8 years	18
Said, 2016	Ages ranged from 18 to 28 years, with a mean of 23.3 years	25
Serra-Renom, 2005	Ages ranged from 22 to 48 years, with a mean of 33 years	45
Siclovan, 2008	NR	45
Stoff-Khalili, 2004	Ages ranged from 17 to 48 years, with a mean of 34 years. The mean body mass index was 21 IU (range, 17-24 IU).	75
Tijerina, 2009	NR	1000
Ventura, 2005	A majority (63) were thin patients with little fatty tissue	100
Yang, 2013	NR	25

BA, breast augmentation. NR, not reported.

### Follow-Up Time

Ten of the included studies did not report a follow-up length; however, several described pleasing long-term results or outcomes. Table 2 summarizes the reported follow-up length for each of these studies.

**Table 2.** Reported Patient Follow-Up of Included Studies

Study	Follow-up period	No. of patients who underwent subfascial BA
Araco, 2007	“Short follow-up period”	511
Aygit, 2013	Ranging from 7 to 28 months (average, 21 months)	27
Barbato, 2004	1 year on average	110
Benito-Ruiz, 2003	NR	16
Brown, 2012	NR	200
Goes, 2003	“Pleasing long-term results”	241
Graf, 2000	NR	8
Graf, 2003	NR	263
Graf, 2005	NR	415
Hunstad, 2010	Ranging from 2 to 24 months	61
Keramidas, 2006	NR	350
Kerfant, 2017	Ranging from 1 to 86 months (average, 22.5 months)	156
Lin Jinde, 2010	NR	10
Munhoz, 2006	The minimum follow-up period was 3 months (average, 16 months; range, 4-45 months)	42
Pereira, 2009	Ranging from 6 months to 3 years	18
Said, 2016	2- to 4-month follow-up period	25
Serra-Renom, 2005	1 day, 1 week, 1 month, 3 months, 1 year	45
Siclovan, 2008	NR	45
Stoff-Khalili, 2004	2.9 years on average (69 of 75 followed up)	75
Tijerina, 2009	NR	1000
Ventura, 2005	NR	100
Yang, 2013	Ranging from 2 to 26 months	25

BA, breast augmentation. NR, not reported.

### Capsular Contracture

**Figure 2.**
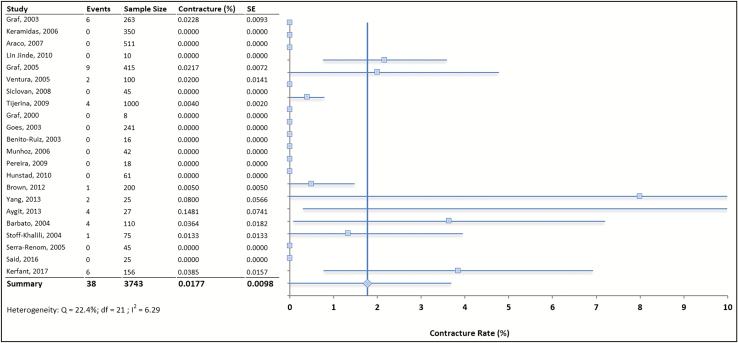
Dot plot depicting the results of the meta-analysis with a distribution of absolute values relative to the line of central tendency (the pooled rate of capsular contraction).

The pooled rate of capsular contracture was 1.01% among all 3743 subfascial augmentations included in this meta-analysis. In each study, the standard error and variability were calculated as well as a weighting for each study (Figure 2). Note studies without capsular contracture hold low weight in the models because of the nature of the mathematical formula used to calculate the weight. The meta-analysis was first conducted by weighting the reported contracture rate for each study and then by modeling the outcomes using a fixed-effects model and a random-effects model with 21 degrees of freedom. The weighted rate of capsular contracture was determined to be 0.69%. A Q-test was applied and a calculated Q-score was found to be 22.4% (α = 0.05) which, when compared to the chi-squared table value of 32.6, was lower and thus suggests that the studies are homogenous in nature. The calculated *I*^2^ value for heterogeneity was extremely low at 6.29% which is also indicative of a low level of heterogeneity across all included studies.^27^ Given low heterogeneity, the fixed-effects model was found to be appropriate for statistical analysis and discussion. Furthermore, a random-effects model was not used, given that a single outcome was investigated and the data were relatively homogenous.

### Outcomes

Among complications, hematoma was reported with a pooled rate of 0.72% overall (Table 3). Rippling was reported in several studies with a pooled rate of 0.24%. The revision rate was similarly low at 0.4% as was the infection rate at 0.1%. There were low rates of hypertrophic scaring (0.18%), sensory deficit (0.24%), malrotation (0.29%), axillary banding (0.21%), asymmetry (0.13%), and seroma (0.10%). The relative complication rate was low for this surgery.

**Table 3.** Summary of Reported Complications

Study	Capsular contracture (%)	Baker class	Hematoma	Ripples	Revisions	Animation	Infection	Hypertrophic scarring	Sensory deficits	Malrotation/ misplacement	Axillary fibrous banding	Asymmetry	Seroma
Araco, 2007	0	0	1	NR	NR	NR	NR	NR	NR	NR	NR	NR	NR
Aygit, 2013	14.81	2	1	0	0	NR	NR	2	NR	0	NR	1 (patient did not request reoperation)	NR
Barbato, 2004	3.64	NR	0	NR	NR	NR	0	2	2	NR	NR	2 (areola asymmetry)	NR
Benito-Ruiz, 2003	0	NR	0	NR	NR	NR	NR	NR	0	NR	Subcutaneous banding at the axilla was noted in 6 patients but disappeared within 3 months of surgery	NR	NR
Brown, 2012	0.5	NR	10	7	8	NR	0	NR	NR	NR	NR	NR	NR
Goes, 2003	0	NR	NR	NR	NR	0	NR	NR	NR	NR	NR	NR	NR
Graf, 2000	0	0	NR	NR	NR	NR	NR	NR	NR	2	NR	NR	NR
Graf, 2003	2.28	2	3	NR	NR	0	0	NR	NR	8	NR	NR	NR
Graf, 2005	2.17	2	5	NR	NR	NR	2	NR	4	NR	3	4	2
Hunstad, 2010	0	0	2	Patient 9.5 Physician 9.6 (both /10)	4	0	0	Patient 9.0 Physician 9.6	Patient 8.8 (on nipple sensitivity)	9.7/10 (10 being none)	NR	Patient: 9.1 Physician: 9.5 (both/10)	NR
Keramidas, 2006	0	NR	1	NR	NR	NR	NR	NR	NR	NR	NR	NR	NR
Kerfant, 2017	3.85	4 class 2; 2 class 3	2	NR	Reoperation rate was 9.94%	0	2	NR	NR	1	NR	0	NR
Lin Jinde, 2010	0	0	0	NR	NR	NR	0	NR	NR	NR	NR	NR	NR
Munhoz, 2006	0	0	0	0	2	NR	0	2	0	NR	5	1	2
Pereira, 2009	0	0	0	NR	NR	0	0	1	NR	NR	NR	NR	0
Said, 2016	0	0	0	NR	NR	NR	NR	NR	NR	0	NR	0	0
Serra-Renom, 2005	0	0	NR	0	0	0	NR	0	NR	0	NR	0	NR
Siclovan, 2008	0	0	NR	NR	NR	0	NR	NR	NR	NR	NR	NR	NR
Stoff-Khalili, 2004	1.33	3	0	1	1	0	0	NR	3	NR	NR	0	0
Tijerina, 2009	0.4	3 and 4	1	0	NR	NR	NR	NR	NR	NR	NR	NR	NR
Ventura, 2005	2	2	0	0	NR	0	0	NR	NR	NR	NR	NR	1
Yang, 2013	8	NR	1	1	NR	NR	0	NR	NR	NR	NR	NR	NR

NR, not reported.

## DISCUSSION

Overall, the systematic review and meta-analysis identified several key findings. Importantly, capsular contracture is a relatively low percentage with a pooled rate of 1.01% and a weighted rate of less than 1%. Our analysis of the heterogeneity allowed us to determine that there was not a significant variability in the studies to throw off the weighted estimate. In contrast, other meta-analysis studies have presented capsular contracture rates as high as 38% for subglandular smooth implants, 15% for submuscular smooth implants, 8.9% for subglandular textured implants, and 8.6% for submuscular textured implants.^34^ Retrospective studies from single practices with large numbers show the lowest rates of capsular contracture for submuscular textured implants at 2.6%^35^ and 7.6%.^36^ These findings suggest that subfascial augmentation has a lower rate of capsular contracture compared with subglandular and perhaps submuscular augmentation.

The authors suppose several factors may contribute to these findings. Notably, subfascial augmentation does not violate the ducts, so this may be a reason why the capsular contraction rates are lower than subglandular augmentation. Others have suggested that cold dissection or blunt dissection may decrease the risk of contracture but this has never been validated. Importantly, the mechanism of animation deformity is that the implant moves as it is under the muscle, and the hope is that subfascial augmentation avoids this outcome. This has yet to be explicitly validated; however, animation deformity is not reported in these studies.

The quality of this review, as with any systematic review, is limited by the studies that were included for analysis. Limitations of our study include the use of solely observational and retrospective studies (Level 4 evidence). The absence of comparative studies, cohort studies, and randomized controls results is an inherent selection bias based on physician preference for candidate patients. In addition, there is an intrinsic risk of bias across many of the included articles primarily stemming from variable length of follow-up and selection of which complications to report. The lack of articles reporting individual patient data, and heterogeneity in outcome measures reporting and scoring further complicate objective conclusions. Standardization of these reporting measures would allow for better analysis of outcomes following treatment.

Unfortunately, the studies presented here lacked key demographic and follow up data. There is simply not enough information on when the patients were followed up to reasonably confirm long-term results. While these results are promising, more studies are needed to adequately power a large cohort. Alternatively, a study comparing interventions with good sample size may help answer that question.

## CONCLUSION

Several studies have proposed a low rate of capsular contracture in subfascial augmentation. This coupled with the benefits of decreased pain, no animation deformity, superior anatomic pocket and blending of the implant junction, subfascial augmentation may prove a superior method in a certain subset of patients. It should be considered in thin or athletic patients. Furthermore, additional comparative, single surgeon studies are needed with appropriate reporting and follow-up to confirm these findings.

## Supplementary Material

ojaa006_suppl_Supplementary_Appendix_A
